# Pulmonary aspiration during procedural sedation for colonoscopy resulting from positional change managed without oral endotracheal intubation

**DOI:** 10.1186/s40981-020-00360-5

**Published:** 2020-07-14

**Authors:** Jun D. Parker

**Affiliations:** 1 Department of Anaesthesia, Portland District Health, 141-151 Bentinck Street, Portland, Victoria, 3305 Australia; 2 School of Clinical Medicine, The University of Queensland, St Lucia, Queensland 4072 Australia

**Keywords:** Sedation, Aspiration, Endoscopy, Airway management, Complications

## Abstract

**Background:**

Pulmonary aspiration under anaesthesia is a feared complication. It is likely that the incidence of aspiration occurring during procedural sedation is underreported; although rare, fatalities do occur. The supine position increases the risk of pulmonary aspiration in gastrointestinal endoscopy during procedural sedation. Immediate oral endotracheal intubation has traditionally been the cornerstone of management for aspiration during anaesthesia; however, this may not be always beneficial when aspiration occurs during procedural sedation. To my knowledge, this is the first case report of aspiration pneumonitis resulting from surgical repositioning during colonoscopy under procedural sedation.

**Case presentation:**

A 72-year-old female underwent elective outpatient diagnostic colonoscopy. Intravenous propofol infusion was commenced for the procedural sedation. A large amount of non-particulate vomitus was expelled from the oropharynx as the patient was repositioned from the left lateral to supine position. Oxygen saturation on pulse oximetry immediately dropped to below 90% during the event. The patient was managed successfully without oral endotracheal intubation.

**Conclusions:**

Anaesthesiologists need to be mindful of factors that raise the risk of aspiration during procedural sedation. Gastrointestinal endoscopy poses a higher risk of aspiration than other procedures, and positional change may be a precipitant. Aspiration that occurs during procedural sedation may be more safely managed by avoiding immediate oral endotracheal intubation.

## Background

Pulmonary aspiration under anaesthesia is a feared complication [1, 2]. It is likely that the incidence of aspiration occurring during procedural sedation is underreported; although rare, fatalities do occur [3]. The supine position increases the risk of pulmonary aspiration in gastrointestinal endoscopy during procedural sedation [3, 4]. Immediate oral endotracheal intubation has traditionally been the cornerstone of management for aspiration during anaesthesia [1]; however, this may not be always beneficial when aspiration occurs during procedural sedation. To my knowledge, this is the first case report of aspiration pneumonitis resulting from surgical repositioning during colonoscopy under procedural sedation. Pulmonary aspiration during procedural sedation may be more safely managed without immediate oral endotracheal intubation.

## Case presentation

A written consent was obtained from the patient for this case report. A 72-year-old female with a body mass index of 29 kg/m^2^ and the American Society of Anesthesiologists physical classification 2 underwent elective outpatient diagnostic colonoscopy for positive faecal occult blood test. The patient fasted for 2 h for clear liquids and 6 h for some limited foods and liquids as per the fasting guidelines by the Australian and New Zealand College of Anaesthetists (ANZCA) [5]. Her past medical history included total abdominal hysterectomy and hypertension managed on amlodipine, irbesartan, and hydrochlorothiazide.

A 22-gauge intravenous catheter was placed in the hand, and standard monitoring as per the sedation guidelines by ANZCA was attached including continuous capnography [6]. Pre-induction vitals were as follows: blood pressure of 111/76 mmHg, pulse rate of 66 beats per minute, oxygen saturation of 98% on room air, and respiratory rate of 18 breaths per minute. The patient was placed in the left lateral position, and a Hudson mask was applied with an oxygen flow rate of 6 L per minute. Intravenous sedation was commenced with propofol with a slow incremental bolus of 80 mg; then, an infusion was commenced at 600 mg per hour (7.9 mg/kg/h). A deep level of sedation was obtained ranging between modified Ramsay Sedation Scale of 5 to 8.

Colonic intubation posed a moderate technical challenge due to an angulated distal sigmoid colon. The patient was repositioned supine 20 min after commencement of the procedure. A size 6.0-mm external diameter nasopharyngeal airway was also placed in the right nostril in anticipation of airway obstruction. Soon after repositioning, a large amount of vomitus consisting of non-particulate yellow fluid was expelled from the mouth. The oropharynx was immediately suctioned with a Yankauer sucker, and the patient was placed in the head-down left lateral position. Oxygen saturation immediately dropped from 97 to 89%. Oxygen flow rate was increased to 10 L per minute, but this did not improve the oxygen saturation beyond 92%. The patient was repositioned supine after noting no further oropharyngeal regurgitation. The sedation level was lightened by reducing the propofol infusion rate to 200 mg per hour (2.6 mg/kg/h) in order for more protective reflexes to return. After several minutes, the patient was again repositioned into the head-down left lateral position at the anaesthesiologist’s request for precautionary reason. Colonoscopy was completed, and the patient was taken to the postoperative recovery unit. Her other observations during the procedure did not show marked variations before and after the aspiration event: the respiratory rate stayed between 20 and 28 breaths per minute, her systolic blood pressure recorded values between 99 and 136 mmHg, and the heart rate ranged between 58 and 72 beats per minute. The total procedure time was 51 min.

After regaining consciousness, the oxygen saturation improved and fluctuated between 94 and 98% with an oxygen flow rate of 6 L a minute through a Hudson mask. The patient reported throat and central chest discomfort, and intravenous paracetamol 1 g and nebulized salbutamol 5 mg were administered. The patient also reported mild dyspnoea, cough, and a sense of pulmonary congestion. Chest X-ray showed no signs of lung consolidation or collapse. Oxygen delivery continued with nasal prongs at 3 L per minute on discharge to the ward.

The patient was admitted overnight due to ongoing oxygen requirement. A spike in temperature of 38.3 °C and right basal crackles were noted in the evening. By the following morning, the patient no longer required oxygen and reported minimal pulmonary symptoms except coughing; therefore, she was discharged home. A follow-up at 5 days showed the patient had completely recovered from the incident.

## Discussion

While much attention has been accorded to aspiration under general anaesthesia [1, 2], aspiration following procedural sedation remains poorly reported in the literature [3]. The incidence of 0.10 to 0.16% has been reported [7, 8]; however, underreporting is likely considering that pulmonary aspiration of gastric content during colonoscopy occurred in 3% of patients assessed with a scintigraphic method [9].

Since Mendelson reported a mortality rate of 3.0% from pulmonary aspiration in 1946 [10], subsequent studies showed varying mortality rates from 4.5% and 6.6% to as high as 70% in at-risk compromised inpatients [11–13]. While data are more limited, mortality rate from aspiration during procedural sedation appears much lower [3].

Traditionally, initial management for aspiration under anaesthesia involved immediate oral endotracheal intubation followed by bronchoscopic suctioning in the case of aspiration of particulate vomitus before application of positive pressure ventilation [1, 2]. Not all cases of aspiration under procedural sedation have been managed with endotracheal intubation [3], and indeed no consensus exists on the recommended management of pulmonary aspiration specifically occurring during procedural sedation. More recently, a role for high-flow nasal oxygen has been suggested [14].

There were several reasons why in this case the patient was not intubated. The morbidity associated with oral endotracheal intubation outweighed the potential benefits. Firstly, the patient aspirated non-particulate matter and bronchoscopic suctioning was not indicated, obviating the need for intubation for this purpose. Secondly, oxygen saturation after aspiration remained adequate. Brief and profound hypoxia in healthy humans is well tolerated in the absence of circulatory compromise [15]. Thirdly, the patient maintained spontaneous breathing, and positive pressure ventilation was not applied. Positive pressure ventilation applied through an oral endotracheal tube even after suctioning could potentially further drive incompletely suctioned aspirates deeper into the smaller airways. The risk of lung contamination in a spontaneously breathing patient may thus be lower than that in a patient under positive pressure ventilation. Fourthly, the procedure did not require general anaesthesia and was of short duration.

Colonoscopy involves colonic insufflation of gas to distend the colon and frequently requires application of abdominal pressure and positional change which may increase the risk of aspiration [16, 17]. Anaesthesiologists and proceduralists both need to be mindful of factors that predispose patients to aspiration during procedural sedation. Coughing during procedural sedation has been suggested as a surrogate marker of impending aspiration with supine positioning further heightening the risk [4]. When prodromal signs of vomiting such as coughing and change in respiratory patterns are evident, prevention of overt aspiration should be initiated immediately. This includes reduction in bowel distension and reduction or cessation in administration of sedatives [18]. Change in body positioning may also be helpful. It is still unclear what constitutes the most protective body positioning. Some advocate sitting or semi-recumbent position [19, 20], while others advocate left lateral position [21] or steep Trendelenburg position [22].

In this case, positioning from left lateral to supine was likely the trigger factor for vomiting and aspiration. A nasopharyngeal airway was also inserted at the corresponding time. An inappropriately long nasopharyngeal airway may irritate the coughing or gag reflex which may predispose the patient to vomiting [23]. Although the length of the nasopharyngeal airway was short, it may have still contributed to the gag reflex.

Prevention of aspiration can be achieved by promoting gastric emptying with pharmacological agents or removing gastric contents with nasogastric intubation before commencement of anaesthesia. Nasogastric intubation may also lead to perioperative aspiration [24], and whether to leave the nasogastric tube in situ or to remove it prior to commencement of anaesthesia remains a matter of contention [25]. Preoperative gastric ultrasound to quantify residual gastric content shows promise in potentially offering preoperative identification of those at risk of pulmonary aspiration. In an Australian study, 4.1% of patients who presented for elective procedure had gastric residual volume at a level considered at risk of pulmonary aspiration [26].

Published management strategies of aspiration during anaesthesia have been generally written for cases requiring general anaesthesia. Aspiration that occurs during procedural sedation may not necessarily benefit from the same approach. While aspiration containing particulate matter should be managed with endotracheal intubation [1], aspiration of non-particulate matter may be more safely managed without immediate oral endotracheal intubation if adequate oxygen saturation is maintained. In a haemodynamically unstable patient following aspiration, endotracheal intubation should be strongly considered as this is a predictor for ventilatory support in the intensive care unit [1, 11]. Developing a guideline specifically for management of aspiration during procedural sedation would be beneficial.

In conclusion, anaesthesiologists need to be mindful of factors that raise the risk of aspiration during procedural sedation. Gastrointestinal endoscopy poses a higher risk of aspiration than other procedures, and positional change may be a precipitant. Aspiration that occurs during procedural sedation may be more safely managed by avoiding immediate oral endotracheal intubation.

## Data Availability

Not applicable.

## Abbreviations

ANZCA: Australian and New Zealand College of Anaesthetists
